# Evaluation of an artificial intelligence-based medical device for diagnosis of autism spectrum disorder

**DOI:** 10.1038/s41746-022-00598-6

**Published:** 2022-05-05

**Authors:** Jonathan T. Megerian, Sangeeta Dey, Raun D. Melmed, Daniel L. Coury, Marc Lerner, Christopher J. Nicholls, Kristin Sohl, Rambod Rouhbakhsh, Anandhi Narasimhan, Jonathan Romain, Sailaja Golla, Safiullah Shareef, Andrey Ostrovsky, Jennifer Shannon, Colleen Kraft, Stuart Liu-Mayo, Halim Abbas, Diana E. Gal-Szabo, Dennis P. Wall, Sharief Taraman

**Affiliations:** 1 CHOC Children’s, Orange, CA USA; 2 University of California, Irvine School of Medicine, Department of Pediatrics, Irvine, CA USA; 3 Bay Area Neuropsychological and Developmental Services, Palo Alto, CA USA; 4 Stanford University, Department of Developmental Behavioral Pediatrics, Lucile Packard Children’s Hospital, Stanford, CA USA; 5 Melmed Center, Scottsdale, AZ USA; 6 Nationwide Children’s Hospital, Columbus, OH USA; 7 The Ohio State University, College of Medicine, Columbus, OH USA; 8 The Nicholls Group, Scottdale, AZ USA; 9 Arizona State University, Department of Psychology, Tempe, AZ USA; 10 University of Missouri, School of Medicine, Columbia, MO USA; 11 Forrest General Hospital, Family Medicine Residency Program, Hattiesburg, MS USA; 12 Hattiesburg Clinic, MediSync Clinical Research, Petal, Hattiesburg, MS USA; 13 Private Practice, Los Angeles, CA, USA; 14 Texas Institute for Neurological Disorders, Frisco, TX USA; 15 Texas Child Neurology, Plano, TX USA; 16 Social Innovation Ventures, Washington, DC USA; 17 Children’s National Health System, Washington, DC USA; 18 Cognoa Inc., Palo Alto, CA USA; 19 Stanford University, Department of Pediatrics, Department of Biomedical Data Science, and Department of Psychiatry and Behavioral Sciences, Stanford, CA USA; 20 Chapman University, Dale E. and Sarah Ann Fowler School of Engineering, Orange, CA USA

**Keywords:** Paediatric research, Diagnosis, Autism spectrum disorders, Paediatric research, Machine learning

## Abstract

Autism spectrum disorder (ASD) can be reliably diagnosed at 18 months, yet significant diagnostic delays persist in the United States. This double-blinded, multi-site, prospective, active comparator cohort study tested the accuracy of an artificial intelligence-based Software as a Medical Device designed to aid primary care healthcare providers (HCPs) in diagnosing ASD. The Device combines behavioral features from three distinct inputs (a caregiver questionnaire, analysis of two short home videos, and an HCP questionnaire) in a gradient boosted decision tree machine learning algorithm to produce either an ASD positive, ASD negative, or indeterminate output. This study compared Device outputs to diagnostic agreement by two or more independent specialists in a cohort of 18–72-month-olds with developmental delay concerns (425 study completers, 36% female, 29% ASD prevalence). Device output PPV for all study completers was 80.8% (95% confidence intervals (CI), 70.3%–88.8%) and NPV was 98.3% (90.6%–100%). For the 31.8% of participants who received a determinate output (ASD positive or negative) Device sensitivity was 98.4% (91.6%–100%) and specificity was 78.9% (67.6%–87.7%). The Device’s indeterminate output acts as a risk control measure when inputs are insufficiently granular to make a determinate recommendation with confidence. If this risk control measure were removed, the sensitivity for all study completers would fall to 51.6% (63/122) (95% CI 42.4%, 60.8%), and specificity would fall to 18.5% (56/303) (95% CI 14.3%, 23.3%). Among participants for whom the Device abstained from providing a result, specialists identified that 91% had one or more complex neurodevelopmental disorders. No significant differences in Device performance were found across participants’ sex, race/ethnicity, income, or education level. For nearly a third of this primary care sample, the Device enabled timely diagnostic evaluation with a high degree of accuracy. The Device shows promise to significantly increase the number of children able to be diagnosed with ASD in a primary care setting, potentially facilitating earlier intervention and more efficient use of specialist resources.

## Introduction

Autism spectrum disorder (ASD) is one of the most common developmental disorders, with a US prevalence of 1.9%^1^. The importance of timely ASD diagnosis, possible as early as 18 months, is underscored by studies linking earlier intervention during the critical neurodevelopmental window to enhanced long-term outcomes. These include greater improvements in social and communication skills^2,3^, cognitive abilities^4,5^, verbal abilities^3,6^, and adaptive behavior^7,8^. Despite documented benefits of early intervention, the mean age of ASD diagnosis in the US remains high at over 4 years^1,9–11^. The estimated three-year delay between initial caregiver concern and an ASD diagnosis is even longer for children who are non-white, female, of lower socioeconomic status, or rural residing^12–15^. Roughly 27% of children with ASD remain undiagnosed at age 8^15^.

One factor contributing to diagnostic delay is the rapid increase in demand for ASD evaluations that has outpaced specialist capacity and led to prolonged wait times^16–19^. Gender, race, and socioeconomic biases in access and diagnosis have also created additional delays for certain subpopulations^1,14^. ASD diagnostic practices in the US are currently fragmented and heavily reliant on a limited number of pediatric subspecialists and team-based behavioral evaluations^20^. These assessments are time-intensive, and families may wait as long as 18 months between initial screening by their healthcare provider (HCP) in the primary care setting and final diagnosis by the specialist^16^.

Low levels of ASD diagnosis in primary care settings present another barrier to timely ASD diagnosis and initiation of interventions. Currently, only ~1% of patients with ASD are diagnosed by primary care HCPs in the US^21,22^. The American Academy of Pediatrics (AAP) recommends that upon a failed ASD screen in primary care, providers comfortable with the Diagnostic and Statistical Manual of Mental Disorders, 5th edition (DSM-5) criteria diagnose ASD or refer to a specialist for further evaluation^18^. Yet, ~60% of children who fail screenings are neither referred to a specialist nor diagnosed in the primary care setting^22^. Common barriers to primary care diagnosis include low confidence in using ASD diagnostic tools due to lack of specialist training and/or lack of time to administer, lack of perceived self-efficacy in making the diagnosis, and lack of time to properly review results with caregivers and discuss treatment recommendations^23–25^. In addition, common screening tools used in primary care settings may miss many cases of ASD. For example, in a cohort of over 20,000 children with ASD outcome data, 61% (278/454) of children who received an outcome diagnosis of ASD screened negative on a common screener^26^.

The complexity of making an accurate ASD diagnostic determination may also add to primary care HCP reluctance to diagnose. ASD has varied clinical presentations and heterogeneous etiology^20^. Additionally, ASD can co-occur with and/or share many overlapping features of other disorders diagnosed in childhood such as attention deficit hyperactivity disorder (ADHD), intellectual disability, speech and language delay, or a variety of psychiatric conditions^27^. Providers are also tasked with making an assessment within a time-constrained clinical encounter where the child may not display behavior characteristic of that seen in the home environment or may become newly behaviorally reactive due to change in environment^28^. These complexities underscore the importance of tailored diagnostic aids to support primary care HCPs in making timely and accurate diagnoses when the diagnosis is straightforward, and to advise further evaluation when the presentation is complex or unclear. However, prior to 2 June 2021, no diagnostic devices had market authorization from the Food and Drug Administration (FDA) to aid in the diagnosis of ASD in the primary care setting^29^.

Existing ASD diagnostic tools^20,30,31^ are used almost exclusively in specialty care settings in the US. Several of these tools, especially when used in combination, have shown good diagnostic accuracy^20^ and consistent performance across trained examiners^32,33^. However, specialized training requirements, the time needed to administer, and insufficient reimbursement rates to justify primary care HCP effort^34^ make them challenging for use in primary care settings. Overdiagnosis can also occur in populations with a lower prevalence of ASD, as is the case in primary care^35^. Reliability may also be reduced if an ASD diagnostic tool intended for combination use is instead used as a stand-alone instrument by time-pressured clinicians^35^. In addition, many of these tools were not designed for remote administration. Geographic and logistical hurdles to finding a trained healthcare professional and appropriate clinical setting may contribute to an imbalance in coverage for some populations^15^.

In order to increase primary care HCP's capacity to promptly diagnose ASD and/or refer complex cases for specialist review, innovative diagnostic aids suitable for use in the primary care setting are urgently needed. Recent research has highlighted the potential for artificial intelligence-based tools to augment various aspects of ASD care^36^. The objective of this study was to test the accuracy of one such tool, an artificial intelligence-based Device that produces recommendations for the HCP after analyzing behavioral features from three distinct inputs: a caregiver questionnaire, an analysis of two short home videos, and an HCP questionnaire.

The Device is a Software as a Medical Device (SaMD)^37^ that deploys a gradient boosted decision tree algorithm. The algorithm uses behavioral features selected through machine-learning techniques as maximally predictive of ASD across a variety of phenotypic presentations^38–43^. The device’s underlying machine learning algorithm was initially developed using patient record data from thousands of children with diverse conditions, presentations, and comorbidities who were either diagnosed with ASD or confirmed not to have ASD based on standardized diagnostic tools and representing both genders across the supported age range. The algorithm was iteratively improved, supplemented with ASD-expert input, and prospectively validated for 7 years prior to this study^38–43^. Use of multimodular Device inputs is consistent with current guidelines and recommendations for an evaluation of ASD that include having both caregiver and clinician input, as well as a structured observation of the child^44^. Previously published analysis of earlier algorithm iterations demonstrated that combining inputs significantly improved the performance of the algorithm^38^.

**Fig. 1:**
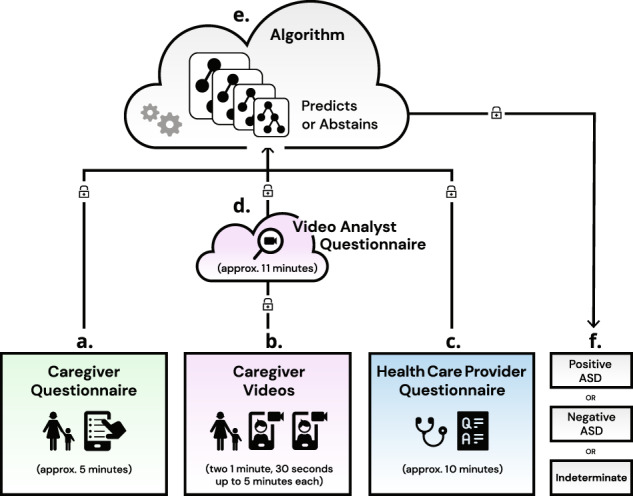
Graphical representation of the Device and its major components. **a** Caregiver uses a smartphone to answer a brief questionnaire in approximately 5 min, **b** Caregiver uploads two short (1 min, 30 s up to 5 min) home videos of their child to be scored by trained video analysts, and **c** their primary care physician (or other qualified healthcare provider) independently answers a short clinical question set in approximately 10 min. These inputs are securely transmitted to the **d** trained analysts where video features are extracted in approximately 11 min. **e** The caregiver, healthcare provider, and video analyst inputs are combined into a mathematical vector for machine-learning analysis and classification. **f** The Device provides an “ASD positive” or “ASD negative” or “no result (indeterminate)” output. The indeterminate output indicates information was insufficiently granular to make a determination at that time.

The Device reports that a subject is positive for ASD, negative for ASD, or “indeterminate” (Fig 1). An indeterminate output is given when Device inputs are insufficiently granular for the algorithm to render a highly predictive output. For example, a patient may exhibit an insufficient number and/or severity of features to be confidently classified by the algorithm as being either ASD negative or ASD positive. The Device’s indeterminate output also referred to in the literature as an “abstention” or “no result” output, is a standard method of risk control in machine-learning algorithms^45,46^. We worked in conjunction with the FDA to establish minimum thresholds for PPV and NPV, which were the primary endpoints for this pivotal study. Once established, we used these values (PPV greater than 65% and NPV greater than 85%) as the boundaries to govern the model’s range of acceptable outcomes during model hyperparameter tuning. We tuned on training/testing data to evaluate combinations of PPV and NPV at or above the FDA thresholds with variable abstention rates. We used cross-validation to maintain the PPV and NPV minimums and to arrive at the current Device abstention thresholds. Abstention in cases of high clinical uncertainty provides a safeguard against known machine-learning failure modes, fosters clinician trust through model transparency, and helps flag cases where additional human expertise or data may be needed^46^. In the primary care setting, and specifically in relation to neurodevelopmental disorders and ASD where symptoms appear on a spectrum and may co-occur with multiple other phenotypically overlapping conditions^27^, ambiguous and complex presentations are expected.

In this double-blinded, active comparator cohort study conducted across six states, we evaluated the ability of the Device to aid in the diagnosis of ASD in children aged 18–72-months for whom a caregiver or HCP had a concern for developmental delay, but were not yet diagnosed. We compared the Device output to the clinical reference standard, consisting of a diagnosis made by a specialist clinician, based on DSM-5 criteria and validated by one or more blinded reviewing specialist clinicians.

## Results

### Subject enrollment

**Fig. 2:**
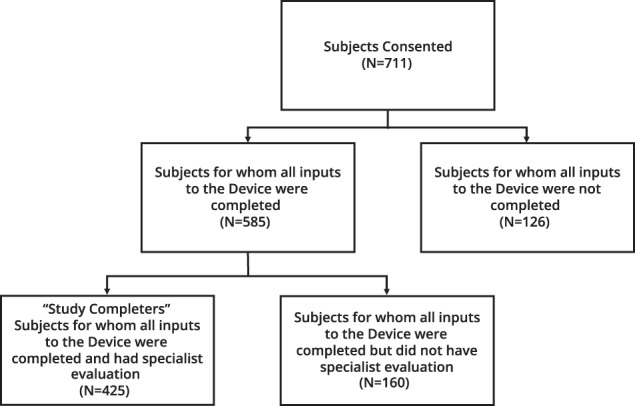
Participant flowchart. A total of 711 participants enrolled in the study between August 2019 and June 2020. Of these participants, 126 dropped out prior to completing all Device inputs. A further 160 dropped out after completing all Device inputs but prior to completing the specialist evaluation. In total, 425 participants completed all Device inputs and the specialist evaluation and were counted as study completers.

A total of 711 participants were enrolled and 425 completed the study between August 2019 and June 2020. Completers and non-completers were similar in terms of demographics (See Table 1). In March 2020, when a national state of emergency was declared in response to COVID-19, a total of 711 participants had enrolled in the study; 585 participants had completed all Device inputs, and 328 of these participants had also completed the specialist evaluation. COVID-19 control measures led to changes in study visit schedules, missed visits, patient discontinuations, and site closures (9 out of 14 sites). Sites that remained open did so with reduced availability to see participants. We estimate that 100 specialist clinician visits could not be completed due to COVID-19. Following the introduction of COVID-19 measures, an additional 97 specialist evaluations were carried out at the sites that remained open. In total, 425 participants completed both the Device input and specialist evaluation component of the study and were included in the final analysis. Data from participants who did not complete both study components were not included in the final analysis. The estimated drop-out rate without the impact of COVID-19 is 26.2% (Fig. 2).

### Baseline characteristics and demographics

#### Participants

The mean age of all study participants was 3.36 years (SD = 1.19) and of completers was 3.33 years (SD = 1.15). The mean age of study completers with ASD was 2.96 years (SD = 1.06) and the mean age of completers for whom the Device rendered an ASD positive result was 2.81 years (SD = 0.94). Additional participant baseline characteristics and demographics are presented in Table 1. Among the study completers, specialist clinicians determined that 61.9% (263/425) had one or more non-ASD developmental or behavioral conditions, 28.7% (122/425) were ASD positive, and 9.4% (40/425) were ASD negative and neurotypical. Table 2 shows all behavioral and developmental concerns for each completer, as listed by caregivers at study commencement, and key comorbidities, as determined by the site specialist as part of their assessment.

**Table 1:** Baseline characteristics and demographics.

Characteristic	Among completers (*N* = 425)	Among all enrolled (*N* = 711)
	% (*n*/*N*)	% (*n*/*N*)
*Age*		
18 months–2 years	13.2% (56/425)	13.6% (97/711)
2–3 years	30.4% (129/425)	28.7% (204/711)
3–4 years	24.2% (103/425)	24.2% (172/711)
4–5 years	21.2% (90/425)	20.7% (147/711)
5 years	11.1% (47/425)	12.8% (91/711)
*Sex*		
Female	36.2% (154/425)	36.1% (257/711)
Male	63.8% (271/425)	62.9% (447/711)
Unknown	0.0% (0/425)	1.0% (7/711)
*Race/ethnicity*		
American Indian only	0.0% (0/425)	0.0% (0/711)
Asian only	4.2% (18/425)	4.4% (31/711)
Black only	13.2% (56/425)	11.4% (81/711)
Hawaiian or Pacific Islander only	0.2% (1/425)	0.4% (3/711)
Hispanic or Latino only	11.5% (49/425)	10.4% (74/711)
Multiple races and/or ethnicities indicated	13.4% (57/425)	13.0% (92/711)
Non-Hispanic White only	53.9% (229/425)	49.0% (349/711)
Other races indicated only	1.2% (5/425)	1.0% (7/711)
Unknown	2.4% (10/425)	10.4% (74/711)
*Parental level of education*		
Some high school	3.1% (13/425)	2.3% (16/711)
High school	10.4% (44/425)	8.4% (60/711)
Some college	22.6% (96/425)	20.1% (145/711)
Associate degree	12.5% (53/425)	11.1% (79/711)
Bachelor’s degree	32.0% (136/425)	30.0% (213/711)
Graduate degree	18.4% (78/425)	18.6% (132/711)
Unknown	1.2% (5/425)	9.3% (66/711)
*Parental income*		
<$25,000	8.7% (37/425)	7.5% (53/711)
$25,000–<$50,000	20.0% (85/425)	17.2% (122/711)
$50,000–<$75,000	19.5% (83/425)	17.7% (126/711)
$75,000–<$100,000	18.8% (80/425)	17.4% (124/711)
$100,000–<$150,000	14.6% (62/425)	14.9% (106/711)
≥$150,000	9.4% (40/425)	8.9% (63/711)
Don't know/not sure/decline to state	8.2% (35/425)	7.6% (54/711)
No record	1.0% (3/425)	8.9% (63/711)

**Table 2:** Participant’s clinical profiles- caregiver and specialist reported.

Developmental delay concerns: caregiver reported		Participant’s clinical profiles as diagnosed by specialist clinicians	
Caregiver concerns	Among completers % (*n*/*N*)	Condition	Among completers % (*n*/*N*)
Prior developmental evaluation	65.0% (160/245)	Language disorder	57.4% (244/425)
Easily frustrated	63.3% (269/425)	Global developmental delay	31.1% (132/425)
Short attention span/distractible	53.4% (227/425)	Attention deficit hyperactivity disorder (ADHD)	24.9% (106/425)
Sensitive to noises/lights/textures	50.0% (212/425)	Phonological disorder	16.0% (68/425)
Impulsive/overactive	48.2% (205/425)	Anxiety disorder	8.2% (35/425)
Doesn’t follow directions	45.2% (192/425)	Oppositional defiant disorder (ODD)	3.8% (16/425)
Requires a lot of parental attention	44.2% (188/425)	Stereotypic movement disorder	3.1% (13/425)
Oppositional/defiant	37.9% (161/425)	Mood disorder	1.9% (8/425)
Overreacts when faced with a problem	37.4% (159/425)	Separation anxiety disorder	1.6% (7/425)
More interested in things than in people	34.3% (146/425)	Intellectual disabilities (ID)	1.4% (6/425)
Poor eye contact	33.0% (140/425)	Learning disorder	1.4% (6/425)
Eats or mouths non-food items	32.7% (139/425)	Selective mutism	0.9% (4/425)
Aggressive	32.5% (138/425)	Other developmental disorder	0.7% (3/425)
Rocking/spinning/hand flapping	29.4% (125/425)	Tic disorder	0.5% (2/425)
Is easily overstimulated in play	27.8% (118/425)	Schizophrenia	0.2% (1/425)
Destructive	27.1% (115/425)		
Need for sameness	27.1% (115/425)		
Unable to separate from the parent	25.6% (109/425)		
Wetting pants/bed	23.8%(101/425)		
Self-injurious (headbangs, bites/hits self)	23.5% (100/425)		
Isolated/withdrawn	21.6% (92/425)		
Difficulty making or keeping friends	21.4% (91/425)		
Obsessions or compulsions	19.1% (81/425)		
Daydreams	18.8% (80/425)		
Bowel accidents	17.4% (74/425)		
Plays with toys abnormally	15.3% (65/425)		
Classroom disruption	15.1% (64/425)		
Excessive worry/fears	15.1% (64/425)		
Individualized education plan	14.8% (63/425)		
Does not show much emotion	8.0% (34/425)		
Psychiatric/emotional problems	4.0% (17/425)		
Sexualized behavior	4.0% (17/425)		
Low self-esteem	3.5% (15/425)		
Sad or depressed	3.1% (13/245)		
Suicidal thoughts	0. (1/425)		

Some participants had multiple caregiver concerns or comorbidities listed thus totals add up to >100%.

#### HCPs

The HCPs who completed input 3 (*n* = 15) were physicians who completed residency training in either general pediatrics or family medicine. They worked across a variety of primary care settings and represented both genders (males 47%). Years in practice post-residency ranged from 1 to 38 with a mean of 16 years in practice. HCPs were not recruited on the basis of any special interest or experience of ASD diagnosis, beyond what was delivered as part of their standard training.

### Device output summary

The Device provided a determinate output (ASD positive or negative) for 31.8% (135/425) of participants. In other words, approximately 1 in 3 subjects received a determinate result from the Device. Of the participants who received an ASD diagnosis by a clinical specialist, 52.5% (64/122) received a determinate result and all were correctly classified by the Device with the exception of a single false negative. Among participants given an ASD negative and neurotypical diagnosis by a clinical specialist, 35.0% (14/40) received an ASD negative Device result and none were misclassified as ASD positive. If the indeterminate safety mechanism were removed, the sensitivity for all study completers would fall to 51.6% (63/122) (95% CI 42.4%, 60.8%) and specificity would fall to 18.5% (56/303) (95% CI 14.3%, 23.3%), due in part to the high number of participants with other neurodevelopmental conditions. Table 3 compares Device output to clinical reference standard diagnosis for all study completers.

**Table 3:** Confusion matrix showing Device output compared to clinical reference standard diagnosis.

Device output	Clinical reference standard output			
	ASD positive	ASD negative, other non-ASD neurodevelopmental condition	ASD negative and neurotypical	Total
ASD positive	63 (14.82%)	15 (3.53%)	−	78 (18.35%)
Indeterminate	58 (13.65%)	206 (48.47%)	26 (6.12%)	290 (68.24%)
ASD negative	1 (0.24%)	42 (9.88%)	14 (3.29%)	57 (13.41%)
Total	122 (28.71%)	263 (61.88%)	40 (9.41%)	425 (100.00%)

### Determinate category analysis

For participants for whom the Device rendered a determinate output, 18.4% (78/425) were classified ASD positive and 13.4% (57/425) ASD negative. The Device yielded these ASD positive or negative results with a PPV of 80.8% (95% CI, 70.3%–88.8%), NPV of 98.3% (95% CI, 90.6%–100%), sensitivity of 98.4% (95% CI, 91.6%–100%), and specificity of 78.9% (95% CI, 67.6%–87.7%).

### Indeterminate category analysis

Specialist diagnosis found 91.0% (264/290) of this group had at least one neurodevelopmental disorder. Specifically, 71.0% (206/290) were ASD negative and had at least one other non-ASD neurodevelopmental or behavioral condition, 20.0% (58/290) were ASD positive, and the remaining 9.0% (26/290) received no developmental delay diagnosis.

### Covariate analysis

Race and ethnicity covariate analysis was based on non-exclusive categories. Therefore, participants who reported multiple races or ethnicities (13.4% of study completers) were included in each race or ethnicity category they identified. While the study was not powered for statistical inference on covariates, we detected no difference in Device performance across participants’ sex, race/ethnicity, income, or education level as determined by examining the overlap of corresponding 95% CIs (see Table 4). The Device provided a higher determinate rate for participants under 3 years old (39%) compared to participants 3 years and over (26%; *p* = 0.006) and higher specificity for participants 3 years and over (88%) compared to participants under 3 years old (67%; *p* = 0.03).

**Table 4:** Device performance metrics by covariate.

Covariate	*n*	PPV %(*n*/*N*)	NPV %(*n*/*N*)	Indeterminate rate %(*n*/*N*)	Sensitivity %(*n*/*N*)	Specificity %(*n*/*N*)
		(95% CI)	(95% CI)	(95% CI)	(95% CI)	(95% CI)
*Age*						
18 months–3 years	185	80% (41/51)	95% (20/21)	61% (113/185)	98% (41/42)	67% (20/30)
Median age (years): 2.67		(67%, 90%)	(76%, 100%)	(54%, 68%)	(87%, 100%)	(47%, 83%)
3–6 years	240	81% (22/27)	100% (36/36)	74% (177/240)	100% (22/22)	88% (36/41)
Median age (years): 4.62		(62%, 94%)	(90%, 100%)	(68%, 79%)	(85%, 100%)	(74%, 96%)
*Sex*						
Female	154	60% (12/20)	96% (24/25)	71% (109/154)	92% (12/13)	75% (24/32)
		(36%, 81%)	(80%, 100%)	(61%, 78%)	(64%, 100%)	(57%, 89%)
Male	271	88% (51/58)	100% (32/32)	67% (181/271)	100% (51/51)	82% (32/39)
		(77%, 95%)	(89%, 100%)	(61%, 72%)	(93%, 100%)	(66%, 92%)
*Race/ethnicity*						
American Indian	4	100% (1/1)	−	75% (3/4)	100% (1/1)	−
		(3%, 100%)		(19%, 99%)	(3%, 100%)	
Asian	27	100% (2/2)	100% (6/6)	70% (19/27)	100% (2/2)	100% (6/6)
		(16%, 100%)	(54%, 100%)	(50%, 86%)	(16%, 100%)	(54%, 100%)
Black	77	95% (19/20)	100% (7/7)	65% (50/77)	100% (19/19)	88% (7/8)
		(75%, 100%)	(59%, 100%)	(53%, 75%)	(82%, 100%)	(47%, 100%)
Hawaiian or Pacific Islander	2	−	−	100% (2/2)	−	−
				(16%, 100%)		
Non-Hispanic White	259	73% (27/37)	97% (37/38)	71% (184/259)	96% (27/28)	79% (37/47)
		(56%, 86%)	(86%, 100%)	(65%, 76%)	(82%, 100%)	(64%, 89%)
Hispanic or Latino (any race)	75	74% (14/19)	100% (10/10)	61% (46/75)	100% (14/14)	67% (10/15)
		(49%, 91%)	(69%, 100%)	(49%, 72%)	(77%, 100%)	(38%, 88%)
Multiple races and/or ethnicities indicated	57	73% (8/11)	100% (8/8)	67% (38/57)	100% (8/8)	73% (8/11)
		(39%, 94%)	(63%, 100%)	(53%, 79%)	(63%, 100%)	(39%, 94%)
No race or ethnicity indicated	10	100% (4/4)	−	60% (6/10)	100% (4/4)	−
		(40%, 100%)		(26%, 88%)	(40%, 100%)	
*Parental level of education*						
Some high school	13	100% (3/3)	−	77% (10/13)	100% (3/3)	−
		(29%, 100%)		(46%, 95%)	(29%, 100%)	
High school	44	91% (10/11)	100% (5/5)	64% (28/44)	100% (10/10)	83% (5/6)
		(59%, 100%)	(48%, 100%)	(48%, 78%)	(69%, 100%)	(36%, 100%)
Some college	96	71% (15/21)	100% (12/12)	66% (63/96)	100% (15/15)	67% (12/18)
		(48%, 89%)	(74%, 100%)	(55%, 75%)	(78%, 100%)	(41%, 87%)
Associate degree	53	78% (7/9)	100% (6/6)	72% (38/53)	100% (7/7)	75% (6/8)
		(40%, 97%)	(54%, 100%)	(58%, 83%)	(59%, 100%)	(35%, 97%)
Bachelor’s degree	136	77% (20/26)	94% (16/17)	68% (93/136)	95% (20/21)	73% (16/22)
		(56%, 91%)	(71%, 100%)	(60%, 76%)	(76%, 100%)	(50%, 89%)
Graduate degree	78	100% (7/7)	100% (16/16)	71% (55/78)	100% (7/7)	100% (16/16)
		(59%, 100%)	(79%, 100%)	(59%, 80%)	(59%, 100%)	(79%, 100%)
*Parental income*						
<$25,000	37	92% (11/12)	100% (2/2)	62% (23/37)	100% (11/11)	67% (2/3)
		(62%, 100%)	(16%, 100%)	(45%, 78%)	(72%, 100%)	(9%, 99%)
$25,000–<$50,000	85	81% (13/16)	100% (10/10)	69% (59/85)	100% (13/13)	77% (10/13)
		(54%, 96%)	(69%, 100%)	(58%, 79%)	(75%, 100%)	(46%, 95%)
$50,000–<$75,000	83	89% (8/9)	91% (10/11)	76% (63/83)	89% (8/9)	91% (10/11)
		(52%, 100%)	(59%, 100%)	(65%, 85%)	(52%, 100%)	(59%, 100%)
$75,000–<$100,000	80	71% (12/17)	100% (12/12)	64% (51/80)	100% (12/12)	71% (12/17)
		(44%, 90%)	(74%, 100%)	(52%, 74%)	(74%, 100%)	(44%, 90%)
$100,000–<$150,000	62	70% (7/10)	100% (13/13)	63% (39/62)	100% (7/7)	81% (13/16)
		(35%, 93%)	(75%, 100%)	(50%, 75%)	(59%, 100%)	(54%, 95%)
> = $150,000	40	67% (2/3)	100% (4/4)	82% (33/40)	100% (2/2)	80% (4/5)
		(9%, 99%)	(40%, 100%)	(67%, 93%)	(16%, 100%)	(28%, 99%)

Severity scores for both the “social communication” and the “restricted and repetitive behavior” categories for all children who received a positive ASD reference diagnosis were recorded by diagnosing specialists per the DSM-5 criteria (Level 3: “Requiring very substantial support”; Level 2: “Requiring substantial support”; Level 1: “Requiring support”). We break these scores down by Device output (positive, indeterminate, negative) in Table 5.

**Table 5:** ASD severity level scores—social communication and restricted and repetitive behavior by device output.

	Device output			
Social communication severity score^1^	Positive ASD	Indeterminate	Negative ASD	Total
1	1	4	−	5
2	18	32	1	51
3	44	21	−	65
Total	63	57	1	121^2^

Restricted and repetitive behavior score^1^	Positive ASD	Indeterminate	Negative ASD	Total
1	1	4	−	5
2	18	32	1	51
3	44	21	−	65
Total	63	57	1	121^2^

^1^In cases where the site specialist scores and the central and reviewing specialist scores differed, the maximum severity score was used.

^2^Due to a data monitoring error, severity scores are only available for 121 of the 122 ASD positive clinical reference standard group.

Level 1 score: “Requiring support”.

Level 2 score: “Requiring substantial support”.

Level 3 score: "Requiring very substantial support”.

Table 6 shows the frequency of key comorbidities across Device outputs.

**Table 6:** Specialist diagnosed comorbidities broken down by device output.

Comorbidity	Device output									
	Positive ASD			Indeterminate			Negative ASD			
	%	*n*	95% CI	%	*n*	95% CI	%	*n*	95% CI	Total
Language disorder	25.4%	62	(20.1%, 31.4%)	62.7%	153	(56.3%, 68.8%)	11.9%	29	(8.1%, 16.6%)	244
Global developmental delay	40.9%	54	(32.4%, 49.8%)	53.8%	71	(44.9%, 62.5%)	5.3%	7	(2.2%, 10.6%)	132
Attention deficit hyperactivity disorder (ADHD)	17.9%	19	(11.1%, 26.6%)	74.5%	79	(65.1%, 82.5%)	7.5%	8	(3.3%, 14.3%)	106
Phonological disorder	4.4%	3	(0.9%, 12.4%)	82.4%	56	(71.2%, 90.5%)	13.2%	9	(6.2%, 23.6%)	68
Anxiety disorder	5.7%	2	(0.7%, 19.2%)	74.3%	26	(56.7%, 87.5%)	20.0%	7	(8.4%, 36.9%)	35
Oppositional defiant disorder (ODD)	6.3%	1	(0.2%, 30.2%)	81.3%	13	(54.4%, 96.0%)	12.5%	2	(1.5%, 38.3%)	16
Stereotypic movement disorder	38.5%	5	(13.9%, 68.4%)	61.5%	8	(31.6%, 86.1%)	0.0%	0	(0.0%, 24.7%)	13
Mood disorder	0.0%	0	(0%, 36.9%)	100.0%	8	(63.1%, 100%)	0.0%	0	(0%, 36.9%)	8
Separation anxiety disorder	0.0%	0	(0%, 41.0%)	100.0%	7	(59.0%, 100%)	0.0%	0	(0%, 41.0%)	7
Intellectual disabilities (ID)	66.7%	4	(22.2%, 95.7%)	33.3%	2	(4.3%, 77.7%)	0.0%	0	(0%, 45.9%)	6
Learning disorder	16.7%	1	(0.4%, 64.1%)	66.7%	4	(22.2%, 95.7%)	16.7%	1	(0.4%, 64.1%)	6
Selective mutism	0.0%	0	(0%, 60.2%)	75.0%	3	(19.4%, 99.4%)	25.0%	1	(0.6%, 80.6%)	4
Other developmental disorder	33.3%	1	(0.8%, 90.6%)	66.7%	2	(9.4%, 99.2%)	0.0%	0	(0%, 70.1%)	3
Tic disorder	0.0%	0	(0%, 84.2%)	100.0%	2	(15.8%, 100%)	0.0%	0	(0%, 84.2%)	2
Schizophrenia	0.0%	0	(0%, 97.5%)	100.0%	1	(2.5%, 100%)	0.0%	0	(0%, 97.5%)	1

### In-person vs remote HCP questionnaire assessment

Of the study completers, 366 (86.1%) of the HCP assessments (Device input 3) were completed via an in-person visit, while 59 (13.9%) were completed via a remote visit. No evidence of performance degradation was found when assessments were performed remotely.

### Time associated with Device use

Completion of input 1 (the caregiver questionnaire) took a median time of 4 min and 56 s. Input 2, completion of video analyst scoring, took a median time of 10 min and 54 s from the time video review began to submission of scores. Input 3 time is self-reported since the HCPs completed a hardcopy of the questionnaire that was only later uploaded to the portal. Qualitatively, HCPs involved in the study reported it took roughly 10 min to complete input 3. Upon completion of inputs, results were immediately produced (Fig 1).

### Diagnostic certainty—agreement amongst clinicians

A diagnosing specialist clinician completed the patient assessment. The patient's case was then independently assessed by a reviewing specialist clinician. If these two specialists disagreed about the ASD diagnosis, then the case was referred to a second *reviewing* specialist. The first reviewing specialist agreed with the diagnosing specialist 79% of the time. The remaining 21% of subjects required a review by a second independent specialist clinician, of which 43% of the time, they agreed with the diagnosing specialist, and 57% of the time, they agreed with the first reviewing specialist. Of the 425 study completers, the diagnosing specialist was either “somewhat” or “completely certain” of the diagnosis 95% of the time. The diagnosing specialist clinician was “completely certain” 67% of the time when diagnosing ASD, 74% of the time when ruling out ASD and suspecting another non-ASD condition, and 95% of the time when ruling out ASD and when the subject was neurotypical (without ASD or other non-ASD condition suspected). In subjects with ASD, when the Device was positive for ASD, the diagnosing specialist clinician was “completely certain” of the diagnosis 71% (95% CI 59–82%) of the time. In subjects with ASD, when the Device abstained from rendering a diagnostic output, the diagnosing specialist clinician was “completely certain” 64% (95% CI 50%–76%) of the time. A test of the significance of the difference between those two proportions returns a *p* value of 0.40.

## Discussion

The Device evaluated in this study was designed to aid HCPs to diagnose ASD in 18–72-month-olds flagged by clinicians or caregivers as having a potential developmental delay. The Device’s user-friendly inputs, timely result provision, and indeterminate output were all designed to maximize its utility, safety, and trustworthiness in the primary care setting. Compared to assessment tools currently available in specialty settings^20,30,31^, the Device tested in this study requires less time to administer and less specialty training. It also captures video data that provides rich insight into the child’s natural behavior outside of the clinic setting. Of note, as a mobile system, the Device is amenable to administration via telemedicine, making it adaptable for use in remote and rural settings as well as during public health emergencies such as the current COVID-19 pandemic.

For nearly a third of this primary care study sample, the Device supported efficient and highly accurate diagnostic evaluations in conjunction with clinical judgment. This is significant since, currently, only ~1% of children with ASD in the US are diagnosed in primary care^21,22^. Of the children for whom the Device made a determinate diagnostic evaluation, 98.4% with ASD received an ASD positive Device result and 78.9% without ASD received an ASD negative Device result. Moreover, 80.8% of children who received an ASD positive Device result were true positives and 98.3% of children with an ASD negative Device result were true negatives. None of the 15 children who received a false-positive Device result were clinically assessed as being neurotypical (without ASD or other non-ASD conditions suspected) and all had a non-ASD developmental-behavioral pediatric condition that could potentially benefit from similar early interventions as ASD. A third of these false-positive cases had one specialist clinician determine that they met diagnostic criteria for ASD. The Device was designed to minimize false negatives to safeguard against missing a diagnosis of ASD, which could have profound consequences such as delayed treatment initiation. There was only a single false negative in this study.

The results of this double-blind active comparator study are promising for future clinical practice and timely in light of the most recent AAP clinical report that advocates for the development of tools to aid in the diagnosis of ASD^18^. Replication and follow-on work are needed, but study results support the potential of the Device to enable a larger portion of children to be diagnosed in a primary care setting than is currently occurring^22^. The Device also has the potential to reduce the mean age of diagnosis and time to diagnosis for a subset of children. For example, the mean age of completers for whom the Device rendered an ASD positive result was 2.81 years, which is 1.5 years earlier than the current average age of diagnosis^1,9,10^. Time burden associated with Device use was also considerably lower than that of existing assessment tools, making it potentially more practical to deploy in time-pressured primary care environments.

The finding of Device performance consistency across sex, race/ethnicity, income, and parental education level is also encouraging, providing preliminary evidence for the Device’s potential to address some well-known ASD diagnostic disparities. When the Device provided a result, for example, it correctly identified 92.3% of girls with ASD. This finding is important given that gender and racial/ethnic biases exist in the current standard of care, such that females, African American, and Hispanic children are less often diagnosed, misdiagnosed more often, and when diagnosed, are diagnosed later on average^47–50^. There was also no evidence of performance degradation when the HCP questionnaire assessments were performed remotely versus in-person. This study finding is reassuring and speaks to the potential for the Device to reduce lags in diagnosis for some vulnerable populations. For example, those who live in rural and remote areas, or low-income families for whom taking time off work to bring their child to in-person assessments may prove challenging.

Although the sample population was ethnically and racially diverse, the study was not powered for statistical inference on covariates such as comorbidities, gender, race/ethnicity, education level, or income level. Future studies that include larger samples of subpopulations are needed to build upon the initial finding of consistent Device performance across these covariates. Formal IQ testing was not conducted as part of the study as a large portion of participants were below the age threshold where non-verbal cognitive abilities can be reliably tested with traditional measures of intelligence^51^. Appropriately powered follow-up studies are needed to confirm Device performance across a range of intellectual levels. Additionally, the Device is currently available only in the English language, and non-English speaking families were excluded from the study. In order to address this bias, multiple language options for non-English speakers are currently in development including Mandarin and Spanish. Compared to the general US population the study cohort also had a higher level of parental education, which is common among clinical trials^52^, and a lower household income. Lower-income may reflect reduced parental participation in the workforce as a result of participants’ developmental delays^53^.

Cost and reimbursement data are also needed to clarify the extent to which the Device could be utilized equitably by primary care HCPs in practice. In light of growing pressures on Medicaid programs to cover the early diagnosis of ASD and concurrent budgetary challenges, better use of existing primary care infrastructure, such as that offered by this Device, may support Medicaid programs to remain financially sustainable while adhering to laws and regulations. Additional costing data are needed, however, to fully understand the likelihood of the Device being widely adopted in US primary care settings, and the extent to which it would be accessible to low-income families or families without health insurance. Additionally, Device use requires access to a smartphone which not all low-income American families may have^54^.

As a risk control measure to minimize the likelihood of false negatives, an indeterminate output that sacrificed coverage was built into the Device. Given the often complex presentation of ASD in primary care settings, including multiple phenotypically overlapping comorbidities and behavioral features that may unfold over time, the finding that the Device abstained from making a recommendation for two out of every three children in this cohort is unsurprising. ASD has degrees of diagnostic complexity that depend on the presentation. Even among experienced specialists, considerable diagnostic uncertainties persist, for example, when diagnosing girls^55^ or children with moderate (vs high or low) levels of observable ASD symptoms^56^. Many children with developmental delays are not autistic or have co-occurring conditions which confound the diagnosis. The drop in sensitivity and specificity that would have been observed in this study were the indeterminate output removed, highlights the importance of abstention as a safety mechanism when deploying AI within complex clinical scenarios^46^. The goal of the Device is not to enable diagnosis of all presentations of ASD, but to aid primary care HCPs to diagnose the subset of children the Device can confidently determine a recommendation for, potentially precluding the need to refer all children for tertiary center evaluation. Reducing tertiary referral loads by even a third could significantly shorten wait times for interventions to begin.

Feasibility data^57^ suggest the indeterminate rate will also vary depending on the makeup of the population the Device is being applied to. In our study population, there was a high rate of complex determinations and a relatively low neurotypical rate (9.4%). In a more neurotypical population, we would anticipate seeing fewer Device abstentions and higher Device specificity. In populations with a higher ASD prevalence than that observed in the study cohort, we would also expect fewer abstentions.

Areas of focus for future research include improving the scalability of the video component of the Device by decreasing reliance on human video analysts in subsequent generations of the algorithm. Research exploring the training and education requirements of HCPs in primary care settings such that they would feel confident using the Device as a diagnostic aid in practice is also needed. Future Device studies currently planned include a registrational study to monitor the stability of Device results as compared to diagnosis over time, including establishing a diagnosis later in children who at the time of initial evaluation did not meet diagnostic criteria for ASD.

Reducing the age of ASD diagnosis and time to diagnosis is essential if early intervention is to commence in the window of brain development where it is most effective. Limited diagnostic capacity in primary care settings, including a lack of tailored diagnostic aids, together with long wait times for specialist evaluations, contribute to current diagnostic bottlenecks. In this double-blind active comparator study of 18–72-month-olds with identified developmental delay concerns, the accuracy of a Device designed to aid HCPs to diagnose ASD was assessed. The AI-based Device was found to facilitate timely and accurate ASD diagnostic evaluation in nearly a third of children in the clinical trial setting while minimizing false negatives across the cohort to maintain clinical safety. While future research is needed, the Device shows the potential to expand primary care diagnostic capacity, thereby enabling earlier intervention for a subset of children and more efficient use of limited specialist resources.

## Methods

This double-blinded active comparator cohort pivotal study was conducted to support the FDA Market Authorization for the Device, a novel ASD diagnosis aid designed for use in primary care settings.

### Clinical trial registration

This study was registered on ClinicalTrials.gov (NCT04151290) prior to study initiation.

### Ethics

The study protocol and informed consent forms were reviewed and approved by a centralized Institutional Review Board (IntegReview IRB). Protocol Number: Q170886. IntegReview IRB granted approval of the study (protocol version 1.0) on 19 July 2019. IntegReview was subsequently purchased by Advarra IRB. Informed consent was obtained from all caregivers whose children participated in the study.

### Recruitment and screening

Children 18–72 months old with identified concerns for developmental delay by an HCP or caregiver were recruited for the study from 14 sites across six US states. Participants represented a population of patients that HCPs would see in their primary care practice. All participants had a caregiver with functional English capability. Caregiver-reported demographic data in the form of participant age, sex, race/ethnicity, and parental education and income were collected to evaluate performance variability among subgroups due to well-documented disparities in ASD diagnosis^14^.

### Device description

The diagnosis aid is a SaMD that uses a machine learning algorithm modeled after standard medical evaluation methodologies. The Device comprises the following: a caregiver-facing mobile application, a video analyst portal, a healthcare provider portal, the underlying machine learning algorithm that drives the Device outputs, and several supporting software and backend services and infrastructure, including privacy and security encryption and infrastructure in compliance with HIPAA and other best practices.

Device interfaces were designed to be user-centric and easy-to-navigate. Relevant Apple, Google, and general Web content accessibility guidelines were followed across all Device interfaces to maximize user inclusivity. Human factors and usability validation testing were conducted throughout the Device design process to ensure external users were able to interact with the Device interfaces without use errors or patterns of use errors that could lead to harm to a patient. Additionally, a risk management process was utilized to identify potential use errors and ensure they were adequately mitigated to a safe and acceptable level. The Caregiver-facing app functions across both iOS and Android platforms. The HCP portal supports the most recent and previous versions of Chrome and Safari browsers running on the most recent and previous versions of Mac and Windows operating systems. The Video Analyst Portal supports the most recent Safari browser running on the most recent iPad operating systems (Fig. 1).

Each Device input has two age-dependent questionnaire versions (18–47 months or 48–71 months). Versions are tailored to reflect developmentally relevant features. The child is automatically assigned the relevant version based on their age. Across the three inputs, each of the two age groups receives 64 questions, however, the breakdown of the number of questions per input varies (number of questions for 18–47-month-olds: input 1 = 18 questions, input 2 = 33 questions, input 3 = 13 questions. Number of questions for 48–71-month-olds: input 1 = 21 questions, input 2 = 28 questions, input 3 = 15 questions). The Device was trained to handle this variation and perform with equal accuracy for both age groups.

Questionnaire content stems from feature ranking experiments conducted previously to identify behavioral, executive functioning, and language and communication features that are maximally predictive of an ASD diagnosis^38–43^. Input questions elicit information about these core behaviors. Behavioral features used by the classifiers vary only slightly between the two age groups. For example, a few questions seek different information in the domains of social interaction and communication in order to best capture the ASD phenotype among children based on developmental trajectories. Core behaviors probed by Device questionnaires are as follows: the ability to integrate different forms of communication, joint attention, pretend play with toys, anger or aggression, language and communication (non-verbal, expressive, receptive and speech), quality of social responses, anxiety level, range and amount of facial expressions, appropriate play, reciprocal communication, appropriateness of eye contact, repetitive mannerisms, creative play, level of engagement, responsive smile, directed gaze, negative response to stimuli, self-injury, group play, obsessive-compulsive, sensory interests, hyperactivity, offering comfort to others, shared interests, imitation, overall developmental challenges/delays, socially directed smile, initiation of activities or interactions, overall quality of interactions with others, unusual interests, interest in others, pretend play with others.

### Study flow

#### Study treatment (ASD assessment using the device)

**Fig. 3:**
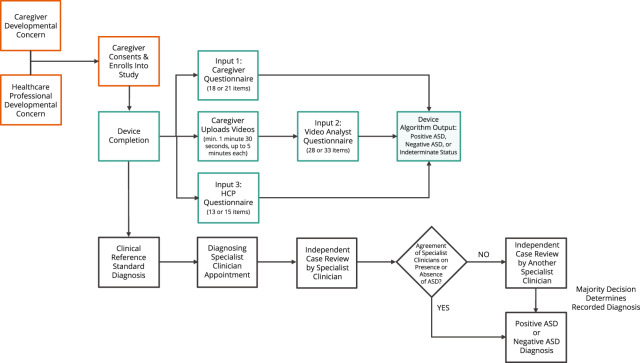
Study flow. Consenting participants meeting inclusion criteria will complete both the Device inputs and the clinical reference standard evaluation.

After providing written informed consent, subject caregivers used the Device Application on their smartphone to complete the caregiver assessment (Device input 1) and record two brief videos of their child (to be used in completion of Device input 2). A HCP completed Device input 3. Results were rapidly available upon completion of the three inputs. The caregivers, video analysts, and HCPs were blinded to each other’s input to the Device and to the Device output (Fig. 3).

- Device input 1: Caregivers completed an 18 or 21-item age-dependent questionnaire via a mobile application.
- Device input 2: Caregivers used the mobile application to upload two, short videos of their child interacting, playing, or talking in natural settings. Videos must be a minimum of 1 min and 30 s, up to 5 min each. The application instructed caregivers on how to take high-quality videos (e.g., showing child’s hands and face with sufficient lighting). In order to ensure strong inter-rater reliability, all video analysts received standardized training, performance analysis, and on-going performance reviews during the study. Prior to receiving video analyst certification, all video analysts were tested on a set of previously unseen training video submissions. These submissions represented a mix of each age group. Analyst performance was required to meet or exceed the operational minimum performance guarantees for PPV, NPV, as well as the abstention rate in order for them to receive certification. In addition to rigorous testing and training standards, all video analysts involved in the study had to meet stringent educational and clinical eligibility requirements. These requirements included: at least a master’s degree from professional backgrounds including psychology, occupational therapy, physical therapy, speech-language pathology, special education, or a related field with specific training in ASD diagnosis and/or treatment, and; at least 5 years of professional and/or clinical experience working with children with ASD. The video analyst scores served as input 2 to the Device.
- Device input 3: A HCP met with the caregiver and child during a 30-min in-person or virtual visit and completed a 13 or 15-item age-dependent questionnaire. Remote visits using a telemedicine platform were held in a manner equivalent to those done in-person, while protecting the subject’s safety and privacy. There was oversight to ensure the methods and conduct of remote assessments were consistent across sites and study subjects to minimize variability in the data.

Time burden associated with Device use was captured electronically for inputs 1 and 2. As HCPs completed a paper version of their questionnaire that was later uploaded to the portal, the time burden associated with input 3 was obtained qualitatively by asking HCPs to estimate the number of minutes questionnaires took to complete. After the caregiver assessment was completed and scorable videos submitted, the caregiver was contacted by a research coordinator to schedule an appointment for a diagnostic evaluation by the diagnosing specialist clinician.

#### Specialist diagnostic assessments

Specialist assessments were conducted by board-certified child and adolescent psychiatrists, child neurologists, developmental-behavioral pediatricians, or child psychologists with more than five years of experience diagnosing ASD. Specialists used structured clinical observation, clinician interview and examination, medical/developmental review, and standardized assessment instruments to provide a diagnosis based on DSM-5 criteria. The structured observational assessment included conversation or free play, symbolic interactive play, and sensory stimulation components. This robust diagnostic process is aligned with best practice recommendations for ASD evaluation^44^. Standardized medical history, physical examination findings, and a video recorded portion of the initial specialist’s assessment were provided to a blinded reviewing specialist clinician who independently evaluated whether DSM-5 criteria for ASD were met. When the first two specialists disagreed, a third specialist was consulted, and the majority decision determined the clinical reference standard diagnosis. Blinded consensus diagnosis introduced an additional level of rigor to the study design, reducing risks associated with confirmation bias, deindividuation, and homogenized group thinking ^35^.

If a specialist clinician: (1) diagnosed co-morbid conditions, (2) determined that the patient was negative for ASD and provided a diagnosis other than ASD, or (3) determined that the patient was neurotypical, those data were also captured. Diagnosis of ASD alone or ASD plus co-morbid condition(s) constituted a positive ASD clinical reference standard diagnosis. Clinicians were also asked to self-report their degree of certainty of the ASD diagnostic conclusion on a Likert scale, with 1 being, “completely uncertain”, 2 being “somewhat uncertain”, 3 being “somewhat certain”, and 4 being “completely certain”.

### Statistical analyses

Statistical analyses were conducted using R 4.0.2 with the PropCIs and Exact packages. Endpoints (PPV, NPV, sensitivity, specificity, and determinate result rate) were evaluated using contingency tables. The selection of predictive values as primary endpoints reflects the utility of the Device in a broad spectrum of subjects, as these values measure how often in the study population the Device correctly identifies a patient with ASD and how frequently it correctly determines that a patient does not have ASD. The Clopper-Pearson interval was used to calculate two-sided 95% CI. The descriptive statistics for sex, race/ethnicity, income, and education were tabulated separately; Device performance was reported for each group and separate two-sided 95% CI were computed. For age analyses, Boschloo’s Exact Test was used to examine statistical differences in performance among subgroups.

### Reporting summary

Further information on research design is available in the Nature Research Reporting Summary linked to this article.

## Supplementary information

Reporting Summary

IRB approval letter

IRB approval_1

## Data Availability

Data are not publicly available because they contain sensitive patient information. Individual, de-identified, participant data that underlie the results reported in this article and study protocol may be made available to qualified researchers upon reasonable request. Proposals should be directed to research@cognoa.com to gain access. Data requestors will be required to sign a data-sharing agreement prior to access. The full study protocol is available on ClinicalTrials.gov.

## Code availability

The code used in the study is proprietary and is not publicly available.
