# Supplementary material for: Evaluation of an artificial intelligence-based medical device for diagnosis of autism spectrum disorder
Source: NPJ Digit Med. 2022 May 5;5:57. doi: 10.1038/s41746-022-00598-6 (PMC9072329; doi:10.1038/s41746-022-00598-6)
Supplement: Supplementary file 1 — Reporting Summary [file 41746_2022_598_MOESM1_ESM.pdf]

nature portfolio

Corresponding author(s): Sharief Taraman

Last updated by author(s): 02/28/2022

Reporting Summary

Nature Portfolio wishes to improve the reproducibility of the work that we publish. This form provides structure for consistency and transparency in reporting. For further information on Nature Portfolio policies, see our Editorial Policies and the Editorial Policy Checklist.

Statistics

For all statistical analyses, confirm that the following items are present in the figure legend, table legend, main text, or Methods section.

n/a	Confirmed	
☐	☒	The exact sample size (*n*) for each experimental group/condition, given as a discrete number and unit of measurement
☒	☐	A statement on whether measurements were taken from distinct samples or whether the same sample was measured repeatedly
☐	☒	The statistical test(s) used AND whether they are one- or two-sided<br>*Only common tests should be described solely by name; describe more complex techniques in the Methods section.*
☐	☒	A description of all covariates tested
☐	☒	A description of any assumptions or corrections, such as tests of normality and adjustment for multiple comparisons
☐	☒	A full description of the statistical parameters including central tendency (e.g. means) or other basic estimates (e.g. regression coefficient) AND variation (e.g. standard deviation) or associated estimates of uncertainty (e.g. confidence intervals)
☒	☐	For null hypothesis testing, the test statistic (e.g. *F*, *t*, *r*) with confidence intervals, effect sizes, degrees of freedom and *P* value noted<br>*Give P values as exact values whenever suitable.*
☒	☐	For Bayesian analysis, information on the choice of priors and Markov chain Monte Carlo settings
☒	☐	For hierarchical and complex designs, identification of the appropriate level for tests and full reporting of outcomes
☒	☐	Estimates of effect sizes (e.g. Cohen's *d*, Pearson's *r*), indicating how they were calculated

Our web collection on statistics for biologists contains articles on many of the points above.

Software and code

Policy information about availability of computer code

Data collection	To maintain confidentiality, subjects will be assigned a unique subject ID number, which will be used on all case report forms and linked to the Informed Consent Forms for audit and verification purposes. All data transmitted and used for analysis will be identified by ID number rather than subject identifying information. Authorized representatives of institutions participating in the study, the Food and Drug Administration (FDA), the Sponsor, and the reviewing IRB and other groups or organizations that have a role in this study will have access to and may view a subject's study information. They will be subject to regulations protecting patient confidentiality (e.g., HIPAA). Data collection will be completed using electronic and/or paper report forms provided by the Sponsor, and data will be recorded into the Sponsor dedicated study data repository. The data will be stored securely on an Amazon S3 cloud server with secured access and SSL encryption. Any data collected on paper will be kept in a secure and locked location at the primary HCP's or specialist clinician's office and periodically transferred to a central research coordinator at each site, before being recorded in an electronic format by the study staff and stored on a HIPAA-secure server.
Data analysis	Statistical analyses were conducted using R 4.0.2 with the PropCIs and Exact packages. The code that supports the findings of this study is proprietary and cannot be publicly disclosed.

For manuscripts utilizing custom algorithms or software that are central to the research but not yet described in published literature, software must be made available to editors and reviewers. We strongly encourage code deposition in a community repository (e.g. GitHub). See the Nature Portfolio guidelines for submitting code & software for further information.

Data

Policy information about availability of data

All manuscripts must include a data availability statement. This statement should provide the following information, where applicable:

- Accession codes, unique identifiers, or web links for publicly available datasets
- A description of any restrictions on data availability
- For clinical datasets or third party data, please ensure that the statement adheres to our policy

Data availability: Data are not publicly available because they contain sensitive patient information. Individual, de-identified, participant data that underlie the results reported in this article and study protocol may be made available to qualified researchers upon request. Proposals should be directed to research@cognoa.com to gain access. Data requestors will be required to sign a data sharing agreement prior to access.The full study protocol is available on ClinicalTrials.gov.

Code Availability: The code used in the study is proprietary, and available only with the permission of the licensors.

Field-specific reporting

Please select the one below that is the best fit for your research. If you are not sure, read the appropriate sections before making your selection.

☐ Life sciences ☒ Behavioural & social sciences ☐ Ecological, evolutionary & environmental sciences

For a reference copy of the document with all sections, see nature.com/documents/nr-reporting-summary-flat.pdf

Behavioural & social sciences study design

All studies must disclose on these points even when the disclosure is negative.

Study description	A double-blinded, multi-site, prospective, active comparator cohort study
Research sample	Female and male subjects between the ages of ≥ 18 months of age and <72 months of age from a general population for whom a caregiver or healthcare provider has a concern about developmental delay. There were 425 study completers, 36% female, 29% ASD prevalence. Mean age of all study completers was 3.33 years (SD = 1.15).
Sampling strategy	The study was conducted in the United States. 14 study sites across 6 states were established. Primary healthcare providers at these sites who identified children at risk for developmental delay or learned of caregiver concern about developmental delay informed caregivers about the clinical study. Caregivers were provided with material(s) describing the study and how to learn more and enroll. Caregivers could enroll in the study by Study Staff, if available at the site, via a website, or via the study research App.
Data collection	After providing written consent, subject caregivers used the Device Application on their smartphone to complete the caregiver assessment (Device Input 1) and record two brief videos of their child (to be used in completion of Device Input 2). A health care provider (HCP) completed Device Input 3. Results were rapidly available upon completion of the three inputs. The caregivers, video analysts, and HCPs were blinded to each other's input to the Device and to the Device output. Specialist assessments were then conducted by board-certified child and adolescent psychiatrists, child neurologists, developmental-behavioral pediatricians, or child psychologists with more than five years of experience diagnosing ASD. Specialists used structured clinical observation, clinician interview and examination, medical/developmental review, and standardized assessment instruments to provide a diagnosis based on DSM-5 criteria. Standardized medical history, physical examination findings, and a video recorded portion of the initial specialists assessment were provided to a blinded reviewing specialist clinician who independently evaluated whether DSM-5 criteria for ASD were met. When the first two specialists disagreed, a third specialist was consulted, and the majority decision determined the clinical reference standard diagnosis.If a specialist clinician: 1) diagnosed co-morbid conditions, or 2) determined that the patient was negative for ASD and provided a diagnosis other than ASD, or 3) determined that the patient was neurotypical, those data were also captured.
Timing	Data collection commenced in August 2019 and finished in June 2020.
Data exclusions	Only data from study completers (those who completed both the investigational Device Inputs and the comparotor specialist diagnostic process) were included in the manuscript analysis. Participant exclusion criteria were established prior to study commencement. These were: Subjects with a prior diagnosis of ASD rendered by a healthcare professional; Subjects with suspected auditory or visual hallucinations or with prior diagnosis of childhood onset schizophrenia; Subjects with deafness or blindness; Subjects with known physical impairments affecting their ability to use their hands; Subjects with major dysmorphic features or prenatal exposure to teratogens (such as fetal alcohol syndrome); Subjects with history, suspicion, or diagnosis of genetic conditions (such as Rett's Syndrome or Fragile X); Subjects with microcephaly; Subjects with history or prior diagnosis of epilepsy or seizures; Subjects with a history of neglect; Subjects with a history of brain malformation, injury or insult requiring interventions such as surgery or chronic medication; Subjects whose age on the date of enrollment is outside the target age range; Subjects or caregivers who have been previously enrolled in any Cognoa clinical study or survey; Subjects whose medical records had been included in any internal Cognoa training or validation sets.
Non-participation	A total of 711 participants were enrolled and 425 completed the study. In March 2020, when a national state of emergency was declared in response to COVID-19. COVID-19 control measures led to changes in study visit schedules, missed visits, patient

	discontinuations, and site closures (9 out of 14 sites). Sites that remained open did so with reduced availability to see participants. We could not acquire full information but estimate that the drop-out rate without the impact of COVID-19 would have been 26.2%
Randomization	None. All subjects will be scheduled to undergo both investigational and comparator diagnosis for ASD.

Reporting for specific materials, systems and methods

We require information from authors about some types of materials, experimental systems and methods used in many studies. Here, indicate whether each material, system or method listed is relevant to your study. If you are not sure if a list item applies to your research, read the appropriate section before selecting a response.

Materials & experimental systems

n/a	Involved in the study
☒	☐ Antibodies
☒	☐ Eukaryotic cell lines
☒	☐ Palaeontology and archaeology
☒	☐ Animals and other organisms
☐	☒ Human research participants
☐	☒ Clinical data
☒	☐ Dual use research of concern

Methods

n/a	Involved in the study
☒	☐ ChIP-seq
☒	☐ Flow cytometry
☒	☐ MRI-based neuroimaging

Human research participants

Policy information about studies involving human research participants

Population characteristics	Female and male subjects between the ages of ≥ 18 months of age and <72 months of age from a general population for whom a caregiver or healthcare provider has a concern about developmental delay were recruited. There were 425 study completers, 36% female, 29% ASD prevalence. Mean age of all study completers was 3.33 years (SD = 1.15). Parental concerns, age of first concern, specialist diagnosed comorbidities and gender, age and race/ethnicity data were collected for all study completers. Data on the caregivers level of education and income was also collected. For all study completers with an ASD positive specialist reference standard DSM-5 ASD severity level scores- social communication and restricted and repetitive behavior- were also collected.
Recruitment	The study was conducted in the United States. 14 study sites across 6 states were established. Primary healthcare providers at these sites who identified children at risk for developmental delay or learned of caregiver concern about developmental delay informed caregivers about the clinical study. Caregivers were provided with material(s) describing the study and how to learn more and enroll. Caregivers could enroll in the study by Study Staff, if available at the site, via a website, or via the study research App.
Ethics oversight	The study protocol and informed consent forms were reviewed and approved by a centralized Institutional Review Board (IntegReview IRB). Protocol Number: Q170886. IntegReview IRB granted approval of study (protocol version 1.0) on July 19, 2019. IntegReview was subsequently purchased by Advarra IRB.

Note that full information on the approval of the study protocol must also be provided in the manuscript.

Clinical data

Policy information about clinical studies

All manuscripts should comply with the ICMJE guidelines for publication of clinical research and a completed CONSORT checklist must be included with all submissions.

Clinical trial registration	This study was registered on ClinicalTrials.gov : NCT04151290
Study protocol	The full study protocol is available on ClinicalTrials.gov
Data collection	The study was conducted in the United States. 14 study sites across 6 states were established. Data collection commenced in August 2019 and finished in June 2020.
Outcomes	We worked in conjunction with the FDA to establish minimum thresholds for PPV and NPV, which were the primary endpoints for this pivotal study (PPV greater than 65% and NPV greater than 85%).
