# Supplementary material for: Evaluation of an artificial intelligence-based medical device for diagnosis of autism spectrum disorder
Source: NPJ Digit Med. 2022 May 5;5:57. doi: 10.1038/s41746-022-00598-6 (PMC9072329; doi:10.1038/s41746-022-00598-6)
Supplement: Supplementary file 2 — IRB approval letter [file 41746_2022_598_MOESM2_ESM.pdf]

INTEGREVIEW IRB

July 19, 2019

Project Manager: Minda Seal, MSA
Sponsor: Cognoa, Inc.
Protocol Number: Q170886
Study Title: "Cognoa ASD Diagnosis Aid Validation Study"

Dear Ms. Seal:

A convened IRB meeting of IntegReview was held on the above-referenced date. The following full board action was taken on initial review of the above-referenced study:

Approved:
Protocol Version 1.0 dated July 11, 2019
"Master" template Informed Consent, English language, dated July 19, 2019

**The IC Acceptance form(s) is being provided to you to allow you the opportunity to review/approve revisions in effort to finalize the "Master" template informed consent(s). Upon IRB approval of Investigators, they will not receive a site-specific informed consent document(s) until IntegReview receives this form(s) and the "Master" template informed consent(s) is finalized. Please review the form for further instructions.**

The Board also reviewed previous study information for the device(s).

**The Board has determined this device to be of non-significant risk.**

**The Board has identified one of these four risk categories that applies to children:**
☒ Clinical investigations not involving greater than minimal risk
☐ Clinical investigations involving greater than minimal risk but presenting the prospect of direct benefit to individual subjects
☐ Clinical investigations involving greater than minimal risk and no prospect of direct benefit to individual subjects, but likely to yield generalizable knowledge about the subjects' disorder or condition
☐ Clinical investigations not otherwise approvable that present an opportunity to understand, prevent, or alleviate a serious problem affecting the health or welfare of children

**ASSENT REQUIREMENTS:**
The board waived assent based on the following:

☒ The children in this study are not capable of providing assent based on age, maturity, and psychological state

**IMPORTANT**

- **The following changes in approved research may not be implemented until you have received approval from IntegReview except where necessary to eliminate apparent immediate hazards to the human subjects:**
  - **Protocol Amendments**
  - **Change in the Principal Investigator and/or Sub-investigators (only if the Sub-investigators will be performing study-related procedures that the PI is not qualified through expertise to perform)**
  - **Change in the address at the study site or the addition of a study site(s)**
- **Only Informed Consent documents containing IntegReview's approval stamp may be utilized:**
  - **There must be procedures in place to guarantee that consent has been voluntarily obtained and properly documented.**
  - **For participants that do not speak English, the informed consent document must be in a language understandable to them; Non-English speaking subjects may not be enrolled until the foreign language Informed Consent Document(s) has been approved by IntegReview.**
  - **Only IntegReview staff may initiate modifications to Informed Consent documents. The Informed Consent document will be maintained in our computer files, and IntegReview will make all revisions following IRB approval**.
  - **Any modifications made to informed consents without prior IRB approval will be considered non-compliance and subject to, but not limited to, full board review, FDA review, suspension and/or termination of IRB approval.**
- **Only recruiting materials containing IntegReview's approval stamp may be utilized. All audio/video recording(s) must be submitted for IRB approval prior to broadcast.**
- **Revision requests should be submitted on IntegReview's forms, which are available in IRBManager.**
- **Visit our website at www.integreview.com for information on research regulations, reporting requirements, Sponsor training, etc.**

IntegReview approval for this study expires **July 18, 2020**.

In order to obtain extended IRB approval, IntegReview must receive your form for continuing review two weeks prior to the IRB expiration date. Appropriate forms will be forwarded to you approximately 4 weeks prior to the approval expiration date. Should the study end before you receive notification, submit a Closure Notification form.

**REPORTING REQUIREMENTS**

To ensure compliance with the applicable federal regulations as well as International Conference on Harmonisation (ICH), E6: Good Clinical Practice: Consolidated Guideline, and/or IntegReview's requirements, notification of the following are required for review/approval:

- **Report immediately:**
  - Findings detected in the monitoring process when those findings could affect the safety of participants or their willingness to continue participation, influence the conduct of the study, or alter IntegReview's approval to continue the study

- Changes in research that were initiated without IRB review and approval to eliminate apparent immediate hazards to the human subjects to ensure the continued safety and welfare of subjects
- Modifications to previously approved documents
- Safety information that may help to provide additional protections for subject's safety and well being, throughout the course of the study and after study completion.
- Communication of results from a research study to subjects when those results directly affect their safety or medical care
- Data Monitoring Committee (DMC/DSMB) Reports

- **Report within 10 calendar days of discovery:**
  - Revisions to the Investigator's Brochure, as applicable
  - Revisions to the report of prior investigations, as applicable
  - Non-compliance – Failure by an investigator and/or sponsor to follow IntegReview's requirements, applicable regulations or to protect human research subjects, including but not limited to the principles of the Belmont Report
  - Serious non-compliance issues – non-compliance as defined as above and as determined to be serious in a way that adversely affects the rights and welfare of human subjects following the investigation and review by the IRB
  - Continuing non-compliance issues – A pattern of repeated non-compliance or serious non-compliance as determined by the IRB
  - Significant deviations – Significant deviations are those that deviate from the approved protocol, informed consent process and affect or potentially affect the safety of subjects. IntegReview does not consider protocol deviations to be different from protocol violations.
  - Unanticipated adverse device effects, as applicable
  - Unanticipated problems should be reported regardless of whether they occur during the study, after the study completion, or after participant withdrawal or completion. Any unanticipated problems involving risks to human subjects or others that are (1) unexpected (in terms of nature, severity, or frequency) given (a) the research procedures that are described in the protocol-related documents, such as the IRB-approved research protocol and informed consent document; and (b) the characteristics of the subject population being studied; (2) related or possibly related to participation in the research (possibly related means there is a reasonable possibility that the incident, experience, or outcome may have been caused by the procedures in the research); and (3) suggests that the research places subjects or others at a greater risk of harm (including physical, psychological, economic, or social harm) than was previously known or recognized. Examples of problems or events that may meet the definition of unanticipated problems involving risk to subjects or others may include, but are not limited to the following:
    - Imminent threat of a reportable event that has not yet occurred
    - Information indicating a change to the risk/benefit ratio of the research
    - Death
    - Breach of confidentiality, including lost or stolen study documents/data
- **Submit prior to publication/distribution:**
  - Any modification(s) to the previously approved Informed Consent document
  - New and/or modifications to previously approved recruiting/miscellaneous materials to be seen or heard by subjects
- **Submit two weeks prior to IntegReview approval expiration date:**
  - Continuing review documents
- **Submit upon completion of the study:**
  - Notification of study closure

At its discretion, IntegReview IRB reserves the right to visit the study site.

IntegReview IRB is organized and operates in accordance with the applicable federal regulations, and ICH Guidelines for Good Clinical Practices, E6. In addition, Standard Operating Procedures have been created to ensure compliance with these regulations and guidelines.

If you have any questions regarding these procedures or if you wish to appeal the decision of the Board, you may address your comments to the IntegReview Chair. Your comments will be reviewed and discussed at the next convened meeting.

**Failure to comply with the Code of Federal Regulations or the requirements or determinations of IntegReview IRB can result in suspension or termination of IntegReview approval**.

Sincerely,

Francine Lopez

Francine Lopez, B.S., CCRP
IRB Coordinator, Co-Chair

IRB Organization #: IORG0000689

**MEMBERSHIP ROSTER**
**As of July 1, 2019**

| | |
|---|---|
| **Sponsor Name:** Cognoa, Inc. | **Protocol Number:** Q170886 |
| **Principal Investigator Name:** | **Meeting Date:** July 19, 2019 |

**C** Denotes consultants (non-voting) assisting in the review of the study when the knowledge and expertise in a particular therapeutic area is not available among the voting members
**A** Denotes members abstaining from the vote
✓ Denotes (voting) members who were in attendance at the meeting and reviewed the study information

| | NAME | DEGREES/ CERTS. | BOARD POSITION | EXPERIENCE | AFFILIATION WITH IRB |
|---|---|---|---|---|---|
| | [M] M. Alexander Kenaston | Ph.D., M.S. (Toxicology), R.N., CIP | Scientific | **IRB Chairperson (Monday)**<br>Toxicology Research Scientist<br>Specializing in First time in human pharmacology studies<br>Licensed Registered Nurse<br>Prior: Project Manager, CRA and CRC for CRO | Non-affiliated Paid Consultant |
| | [M] Jami Brackeen | CST, CCRP | Scientific | **IRB Co-chair and Coordinator (Monday)**<br>Prior: Quality Control Associate for IRB Regulatory Compliance; Certified Surgical Technologist | Full time Employee |
| | [M] Olga Obrda | B.S. (Chemistry) | Scientific | Prior: Project Management and Business Development for CRO | Non-affiliated Paid Consultant |
| | [M] Ashley Hutson | | Non-scientific | Patient advocate for Spinal Cord Injuries<br>Experience volunteering as research subject in clinical trials | Non-affiliated Paid Consultant |
| | [M] Karen Haslund | M.D. | Scientific | Board certified physician, Pediatrics | Non-affiliated Paid Consultant |

[M] Denotes regular Monday board members [T] Denotes regular Tuesday board members [W] Denotes regular Wednesday board members [Th] Denotes regular Thursday board members
[F] Denotes regular Friday board members Other members are alternates All regular Board members can serve as alternates on other Boards as specified by their positions (e.g. Scientific for Scientific)
Non-scientific members represent the general perspective of study participants
**Note: In addition to our regular members, we have access to specialists in therapeutic areas not represented on this roster.**

| | |
|---|---|
| **Sponsor Name:** Cognoa, Inc. | **Protocol Number:** Q170886 |
| **Principal Investigator Name:** | **Meeting Date:** July 19, 2019 |

| | NAME | DEGREES/ CERTS. | BOARD POSITION | EXPERIENCE | AFFILIATION WITH IRB |
|---|---|---|---|---|---|
| | [M] Bryson Michael Duhon | Pharm.D., BCPS | Scientific | Clinical Assistant Professor UT College of Pharmacy<br>Adjunct Professor at UT Health Science Center (UTHSCSA) Department of Medicine | Non-affiliated Paid Consultant |
| | [T] Charles F. Ryan | Ph.D., M.S. (Pharmacology & Toxicology), R.Ph. | Scientific | **IRB Chairperson (Tuesday)**<br>Specializing in First time in human studies<br>Pharmacology & Toxicology<br>Radiation Safety/Radioisotope<br>Nutritional/Food supplements and Medical Foods | Non-affiliated Paid Consultant |
| | [T] Tonya Reed | | Non-scientific | **IRB Co-Chair and Coordinator (Tuesday)**<br>Prior: Project Assistant for CRO | Full time Employee |
| | [T] Sara Bartos | M.D. | Scientific | Board certified physician, Internal Medicine | Non-affiliated Paid Consultant |
| | [T] Marcy Goodfleisch | B.S., M.A. (Liberal Studies); Mediator (Civil & Family Dispute Resolution); Graduate work in Communications and English | Non-scientific | Adjunct University Professor<br>Ethicist<br>Management & Communication Consultant<br>Prior: Clinic Administrator for nationally recognized HIV Clinic and large FQHC community health center. | Non-affiliated Paid Consultant |
| | [T] Christine du Castel | M.D. | Scientific | Medical Advisor; previously licensed to practice General Medicine in France | Non-affiliated Paid Consultant |

[M] Denotes regular Monday board members [T] Denotes regular Tuesday board members [W] Denotes regular Wednesday board members [Th] Denotes regular Thursday board members
[F] Denotes regular Friday board members Other members are alternates All regular Board members can serve as alternates on other Boards as specified by their positions (e.g. Scientific for Scientific)
Non-scientific members represent the general perspective of study participants
**Note: In addition to our regular members, we have access to specialists in therapeutic areas not represented on this roster.**

INTEGREVIEW IRB

**IRB Organization #: IORG0000689**

| | |
|---|---|
| **Sponsor Name:** Cognoa, Inc. | **Protocol Number:** Q170886 |
| **Principal Investigator Name:** | **Meeting Date:** July 19, 2019 |

| | NAME | DEGREES/ CERTS. | BOARD POSITION | EXPERIENCE | AFFILIATION WITH IRB |
|---|---|---|---|---|---|
| ✓ | [T] Michael D. Aldridge | Ph.D. (Nursing Education), R.N., CNE | Scientific | Assistant Professor of Nursing<br>Prior: various Nursing roles for pediatric intensive care unit, including Specialty Education Coordinator for pediatric ICU, previous experience as an IRB member for Institutional IRB | Non-affiliated Paid Consultant |
| | [W] Carolyn Hensler | B.S. (Physical Education) | Scientific | **IRB Chairperson (Wednesday)**<br>Quality Assurance and Quality Control Administrator for CRO<br>Prior: Clinical Research Monitor; and Project Manager | Non-affiliated Paid Consultant |
| | [W] Melanie Castillo | | Non-scientific | **IRB Co-Chair and Coordinator (Wednesday)**<br>Prior: Quality Assurance and Clinical Research Coordinator for clinical research site | Full time Employee |
| | [W] Raymond Carr | R.Ph. | Scientific | Staff Pharmacist | Non-affiliated Paid Consultant |
| | [W] Christopher P. Martin | Pharm.D., M.S., BCPS | Scientific | Clinical Assistant Professor, Division of Pharmacotherapy UT; Clinical Pharmacy Coordinator; Assistant Professor University of Oklahoma Health Sciences Center, College of Pharmacy | Non-affiliated Paid Consultant |
| | [W] Robert A. Blum | Pharm.D. | Scientific | Prior: Principal investigator for Phase I-IV research | Non-affiliated Paid Consultant |
| | [W] William K. Rawlinson | M.D., FCCP, FAASM | Scientific | Board certified physician, Pulmonary Disease, Critical Care and Internal Medicine | Non-affiliated Paid Consultant |

[M] Denotes regular Monday board members [T] Denotes regular Tuesday board members [W] Denotes regular Wednesday board members [Th] Denotes regular Thursday board members
[F] Denotes regular Friday board members Other members are alternates All regular Board members can serve as alternates on other Boards as specified by their positions (e.g. Scientific for Scientific)
Non-scientific members represent the general perspective of study participants
**Note: In addition to our regular members, we have access to specialists in therapeutic areas not represented on this roster.**

IRB Organization #: IORG0000689

| **Sponsor Name:** Cognoa, Inc. | **Protocol Number:** Q170886 |
|---|---|
| **Principal Investigator Name:** | **Meeting Date:** July 19, 2019 |

| | NAME | DEGREES/ CERTS. | BOARD POSITION | EXPERIENCE | AFFILIATION WITH IRB |
|---|---|---|---|---|---|
| | [Th] Frederick Kopec | J.D., B.A. (Philosophy) | Non-scientific | **IRB Chairperson (Thursday)**<br>Licensed: Practice of Law<br>Ethicist | Non-affiliated Paid Consultant |
| | [Th] Bridget Briseno | | Non-scientific | **IRB Co-Chair and Coordinator (Thursday)**<br>Prior: Project Assistant for Phase I facility | Full time Employee |
| | [Th] Michael Romain | M.D. | Scientific | Board certified physician, Internal Medicine | Non-affiliated Paid Consultant |
| | [Th] Mary O'Connell | | Scientific | Quality & Regulatory Affairs Manager at CRO<br>Prior: Clinical Research Recruiter, Data Associate, Coordinator and QC Auditor for CRO<br>IRB Administrator<br>Emergency Medical Technician Paramedic | Non-affiliated Paid Consultant |
| | [Th] Matthew Pfeiffer | Ph.D. (Pharmacology & Toxicology) | Scientific | Project Manager and CRA for CRO<br>Specializing in human studies; Pharmacology, Toxicology, CNS, Infectious disease, Metabolic/Endocrine Disorders, Oncology | Non-affiliated Paid Consultant |
| | [Th] Susan Parker Ginnings | R.Ph. | Scientific | Prior: Hospital Pharmacy Supervisor | Non-affiliated Paid Consultant |
| ✓ | [F] Mary Ruwart | Ph.D. (Biophysics); B.S. (Biochemistry) | Scientific | **IRB Chairperson (Friday)**<br>Research Scientist<br>Specializing in Drug Delivery Systems, Diabetes, GI Diseases, Drug Metabolism | Non-affiliated Paid Consultant |
| ✓ | [F] Francine Lopez | B.S., CCRP | Non-scientific | **IRB Co-chair and Coordinator (Friday)**<br>Prior: Managed Phase I-IV clinical research site | Full time Employee |

[M] Denotes regular Monday board members [T] Denotes regular Tuesday board members [W] Denotes regular Wednesday board members [Th] Denotes regular Thursday board members
[F] Denotes regular Friday board members Other members are alternates All regular Board members can serve as alternates on other Boards as specified by their positions (e.g. Scientific for Scientific)
Non-scientific members represent the general perspective of study participants
**Note: In addition to our regular members, we have access to specialists in therapeutic areas not represented on this roster.**

IRB Organization #: IORG0000689

| | |
|---|---|
| **Sponsor Name:** Cognoa, Inc. | **Protocol Number:** Q170886 |
| **Principal Investigator Name:** | **Meeting Date:** July 19, 2019 |

| | NAME | DEGREES/ CERTS. | BOARD POSITION | EXPERIENCE | AFFILIATION WITH IRB |
|---|---|---|---|---|---|
| ✓ | [F] Dennis Brannon | R.Ph., B.S. (Animal Science) | Scientific | Clinical Research Consultant, Director of Pharmacy; Executive Director Clinical Development<br>Prior: Senior Project Manager and CRA for CRO | Non-affiliated Paid Consultant |
| ✓ | [F] Laurajo Ryan | Pharm.D., MSc (Clinical Investigations), BCPS, CDE | Scientific | Clinical Associate Professor of Pharmacotherapy UT Austin, Department of Medicine UTHSCSA, Clinical Pharmacist Specialist South Texas Veterans Administration | Non-affiliated Paid Consultant |
| ✓ | [F] Bennie C. Lopez | MBA | Non-scientific | Adult and juvenile prisoner population representative<br>Teacher with Austin ISD Alternative Learning Center<br>Prior: Corrections Officer and retired military | Non-affiliated Paid Consultant |
| ✓ | [F] Patricia M. Houser | M.D. | Scientific | Board certified physician; Family practice | Non-affiliated Paid Consultant |
| | Dawn East | R.N., BSN (Nursing), ONC. | Scientific | Executive management experience in Oncology for 30+ years at multiple CROs and Site networks. | Non-affiliated Paid Consultant |
| | Christina H. Walker | M.D., B.S. | Scientific | Physician: Family Medicine [Sports Medicine] | Non-affiliated Paid Consultant |
| | Yessica Chapa | M.D. | Scientific | Board certified physician; Family practice | Non-affiliated Paid Consultant |
| | Kate D. Jeffers | Pharm.D., MHA, BCOP | Scientific | Board certified Oncology Pharmacist | Non-affiliated Paid Consultant |
| | Victoria Govea | CCRP | Non-scientific | Director of IRB Operations<br>Prior: IRB Co-chair and Coordinator | Full time Employee |

[M] Denotes regular Monday board members [T] Denotes regular Tuesday board members [W] Denotes regular Wednesday board members [Th] Denotes regular Thursday board members
[F] Denotes regular Friday board members Other members are alternates All regular Board members can serve as alternates on other Boards as specified by their positions (e.g. Scientific for Scientific)
Non-scientific members represent the general perspective of study participants
**Note: In addition to our regular members, we have access to specialists in therapeutic areas not represented on this roster.**

INTEGREVIEW IRB

**IRB Organization #: IORG0000689**

| | |
|---|---|
| **Sponsor Name:** Cognoa, Inc. | **Protocol Number:** Q170886 |
| **Principal Investigator Name:** | **Meeting Date:** July 19, 2019 |

| | NAME | DEGREES/ CERTS. | BOARD POSITION | EXPERIENCE | AFFILIATION WITH IRB |
|---|---|---|---|---|---|
| | Lynn Goldman | B.S. (Nutrition), MSHP (Healthcare Administration), RD, LD, CCRP | Scientific | IRB Operations Manager<br>Prior: IRB Co-chair and Coordinator<br>Research Coordinator, Health Care Administration and Education, Clinical Nutrition, Registered Dietician/Certified Pediatric Nutrition Specialist | Full time Employee |
| | Rosa S. Sandoval | B.S. (Chemistry), CCRP | Scientific | IRB Operations Supervisor<br>Prior: Senior IRB Co-chair and Coordinator<br>Clinical Research Coordinator and Medical Research Assistant for University | Full time Employee |
| | Levi Machado | CCRP | Non-scientific | IRB Training Administrator<br>Prior: IRB Co-chair and Coordinator | Full time Employee |
| | Angelica Martinez | CCRP | Non-scientific | IRB Operations Specialist<br>Prior: IRB Co-Chair and Coordinator | Full time Employee |

[M] Denotes regular Monday board members [T] Denotes regular Tuesday board members [W] Denotes regular Wednesday board members [Th] Denotes regular Thursday board members
[F] Denotes regular Friday board members Other members are alternates All regular Board members can serve as alternates on other Boards as specified by their positions (e.g. Scientific for Scientific)
Non-scientific members represent the general perspective of study participants
**Note: In addition to our regular members, we have access to specialists in therapeutic areas not represented on this roster.**
