# Supplementary material for: Evaluation of an artificial intelligence-based medical device for diagnosis of autism spectrum disorder
Source: NPJ Digit Med. 2022 May 5;5:57. doi: 10.1038/s41746-022-00598-6 (PMC9072329; doi:10.1038/s41746-022-00598-6)
Supplement: Supplementary file 3 — IRB approval_1 [file 41746_2022_598_MOESM3_ESM.pdf]

Note to File Re: Statement of Investigator, Form FDA 1572

March 1, 2021

Dear Customer,

As previously announced, Advarra, Inc. ("Advarra") acquired IntegReview Ltd. ("IntegReview") effective November 9, 2020.

On May 28, 2021, IntegReview will de-activate its IORG registration (IORG0000689). All ongoing studies under IntegReview's IRB oversight will be transferred to Advarra IRB oversight at that time, and all studies will fall under the Advarra IRB registration number IORG0000635.

The FDA does not require that an updated or new 1572 be completed to reflect this change in information; however, FDA recommends that the investigator document the change in the clinical study records and inform the sponsor of these changes.[1] This Note to File may serve as this documentation.

The following addresses should be used for new 1572s:

**Advarra IRB Review**	**Address**
Studies reviewed by the US Board	6100 Merriweather Drive, Suite 600<br>Columbia, MD 21044
Studies reviewed by the Canadian Board	300-372 Hollandview Trail<br>Aurora ON L4G 0A5

We look forward to continuing our relationship with your company.

Sincerely,

Michele Russell-Einhorn
Chief Compliance Officer and Institutional Official, Advarra

[1] *Information Sheet Guidance for Sponsors, Clinical Investigators, and IRBs, Frequently Asked Questions – Statement of Investigator (Form FDA 1572)*, May 2010, available at https://www.fda.gov/downloads/RegulatoryInformation/Guidances/UCM214282.pdf
